# The Opioid Epidemic and Primary Headache Disorders: A Nationwide Population-Based Study

**DOI:** 10.7759/cureus.9743

**Published:** 2020-08-14

**Authors:** Urvish K Patel, Preeti Malik, Dhaivat Shah, Ashish Sharma, Jatminderpal Bhela, Bindi Chauhan, Deepkumar Patel, Nashmia Khan, Ashish Kapoor, Tapan Kavi

**Affiliations:** 1 Neurology and Public Health, Icahn School of Medicine at Mount Sinai, New York, USA; 2 Public Health, Icahn School of Medicine at Mount Sinai, New York, USA; 3 Pediatrics, The Children's Hospital at Montefiore, Bronx, USA; 4 Clinical Research, Icahn School of Medicine at Mount Sinai, New York, USA; 5 Internal Medicine, Yuma Regional Medical Center, Yuma, USA; 6 Psychiatry, MetroHealth System, Case Western Reserve University School of Medicine, Cleveland, USA; 7 Public Health, Long Island University, New York, USA; 8 Public Health, School of Health Sciences and Practice, New York Medical College, Valhalla, USA; 9 Internal Medicine, MultiCare Tacoma General Hospital, Tacoma, USA; 10 Neurology, Bayonne Medical Center - CarePoint Health & Jersey City Medical Center - RWJBarnabas Health, Jersey City, USA; 11 Neurology, Cooper Neurological Institute, Cooper University Hospital, Camden, USA

**Keywords:** opioid, opioid epidemic, headache, migraine, cluster headache, tension headache, primary headache disorder, nationwide inpatient sample

## Abstract

Introduction

The opioid epidemic has been linked to several other health problems, but its impact on headache disorders has not been well studied. We performed a population-based study looking at the prevalence of opioid use in headache disorders and its impact on outcomes compared to non-abusers with headaches.

Methodology

We performed a cross-sectional analysis of the Nationwide Inpatient Sample (years 2008-2014) in adults hospitalized for primary headache disorders (migraine, tension-type headache [TTH], and cluster headache [CH]) using the International Classification of Diseases, Ninth Revision, Clinical Modification (ICD-9-CM) codes. We performed weighted analyses using the chi-square test, Student’s t-test, and Cochran-Armitage trend test. Multivariate survey logistic regression analysis with weighted algorithm modelling was performed to evaluate morbidity, disability, and discharge disposition. Among US hospitalizations during 2013-2014, regression analysis was performed to evaluate the odds of having opioid abuse among headache disorders.

Results

A total of 5,627,936 headache hospitalizations were present between 2008 and 2014 of which 3,098,542 (55.06%), 113,332 (2.01%), 26,572 (0.47%) were related to migraine, TTH, and CH, respectively. Of these headache hospitalizations, 128,383 (2.28%) patients had abused opioids. There was a significant increase in the prevalence trend of opioid abuse among patients with headache disorders from 2008 to 2014. The prevalence of migraine (63.54% vs. 54.86%), TTH (2.29% vs. 2.01%), and CH (0.59% vs. 0.47%) was also higher among opioid abusers than non-abusers (p<0.0001). Opioid abusers with headaches were more likely to be younger (43 years old vs. 50 years old), men (30.17% vs. 24.78%), white (80.83% vs. 73.29%), Medicaid recipients (30.15% vs. 17.03%), and emergency admissions (85.4% vs. 78.51%) as compared to opioid non-abusers with headaches (p<0.0001). Opioid abusers with headaches had higher prevalence and odds of morbidity (4.06% vs. 3.70%; adjusted odds ratio [aOR]: 1.48; 95% CI: 1.39-1.59), severe disability (28.14% vs. 22.43%; aOR: 1.58; 95% CI: 1.53-1.63), and discharge to non-home location (17.13% vs. 18.41%; aOR: 1.35; 95% CI: 1.30-1.40) as compared to non-abusers. US hospitalizations in years 2013-2014 showed the migraine (OR: 1.61; 95% CI: 1.57-1.66), TTH (OR: 1.43; 95% CI: 1.22-1.66), and CH (OR: 1.34; 95% CI: 1.01-1.78) were linked with opioid abuse.

Conclusion

Through this study, we found that the prevalence of migraine, TTH, and CH was higher in opioid abusers than non-abusers. Opioid abusers with primary headache disorders had higher odds of morbidity, severe disability, and discharge to non-home location as compared to non-abusers.

## Introduction

Headache disorder is one of the leading conditions for emergency department visit, accounting for 0.5% to 2.8% of all visits [1,2]. Headaches are also the third highest cause worldwide of years lost due to disability [3]. Migraine, tension-type headache (TTH), and cluster headache (CH) are the most common types of primary headache. Prolonged personal suffering, impaired quality of life, and economic burden are commonly associated with chronic headache disorders [4].

Opioids are often prescribed more than any other non-steroidal anti-inflammatory drugs as a primary level of therapy for headache [5]. Prolonged use of an opioid in patients with headache disorders, such as migraine, carries a high risk of medication overuse headache. According to the American Headache Society, prolonged and overuse of opioids (more than 10 times per month) can lead to medication overuse headaches and cause occasional migraines to transition to chronic migraine [6]. The opioid epidemic has affected a number of other disorders; however, its impact on headache disorders has not been well studied. The relationship is especially important as opioids are an important treatment modality for headache disorders.

Hence, we conducted this study to determine the trend of opioid abuse among patients with primary headache disorders, evaluate whether opioid-dependent and non-dependent opioid abuse associated with headache disorders, and its relationship with outcomes like morbidity, disability, and discharge disposition.

## Materials and methods

Data was obtained from the Agency for Healthcare Research and Quality's Healthcare Cost and Utilization Project (HCUP) Nationwide Inpatient Sample (NIS) between January 2008 and December 2014. The NIS is the largest publicly available all-payer inpatient care database in the United States and contains discharge-level data provided by states that participate in the HCUP (including a total of 46 in 2011). This administrative dataset contains data on approximately eight million hospitalizations in 1,000 hospitals that were chosen to approximate a 20% stratified sample of all US community hospitals, representing more than 95% of the national population. Criteria used for stratified sampling of hospitals into the NIS include hospital ownership, patient volume, teaching status, urban or rural location, and geographic region. Discharge weights are provided for each patient discharge record, which allows extrapolation to obtain national estimates. Each hospitalization is treated as an individual entry in the database and is coded with one principal diagnosis, up to 24 secondary diagnoses, and 15 procedural diagnoses associated with that stay. Detailed information on NIS is available at http://www.hcup-us.ahrq.gov/db/nation/nis/nisdde.jsp. The NIS is a de-identified database, so informed consent or Institutional Review Board approval was not needed for the study. The HCUP Data Use Agreement (HCUP-348L73IZS) for the data utilized in this study was obtained.

Study population

We used the International Classification of Diseases, Ninth Revision, Clinical Modification (ICD-9-CM) codes to identify adult patients admitted to hospital with a primary diagnosis of migraine, TTH, and CH (ICD-9-CM code Migraine: 346, TTH: 339.1 or 307.81, and CH: 339.0). Similarly, patients with opioid dependence and non-dependent opioid abuse were identified using ICD-9-CM code 304.0 and 305.5, respectively. Age <18 years and admissions with missing data for age, gender, and race were excluded. The sample size was based on the available data. Data from NIS has previously been used to identify and analyze the trends, outcomes, healthcare costs, and disparities of care [7,8]. We have not considered available NIS data from the years 2015 and 2016 due to the lack of literature showing the validity of ICD-10 for identifying headache disorders.

Patient and hospital characteristics

Patient characteristics of interest were age, gender, race, insurance status, and concomitant diagnoses as defined above. Race was defined by white (referent), African American, Hispanic, Asian or Pacific Islander, and Native American. Insurance status was defined by Medicare (referent), Medicaid, private insurance, and other/self-pay/no charge. We defined the severity of comorbid conditions using Deyo's modification of the Charlson comorbidity index (CCI) (Table 1).

**Table 1:** Deyo’s modification of CCI CCI, Charlson comorbidity index; ICD-9-CM, International Classification of Diseases, Ninth Revision, Clinical Modification

Condition	ICD-9-CM codes	Charlson score
Myocardial infarction	410–410.9	1
Congestive heart failure	428–428.9	1
Peripheral vascular disease	433.9, 441–441.9, 785.4, V43.4	1
Cerebrovascular disease	430–438	1
Dementia	290–290.9	1
Chronic pulmonary disease	490–496, 500–505, 506.4	1
Rheumatologic disease	710.0, 710.1, 710.4, 714.0–714.2, 714.81, 725	1
Peptic ulcer disease	531–534.9	1
Mild liver disease	571.2, 571.5, 571.6, 571.4–571.49	1
Diabetes	250–250.3, 250.7	1
Diabetes with chronic complications	250.4–250.6	2
Hemiplegia or paraplegia	344.1, 342–342.9	2
Renal disease	582–582.9, 583–583.7, 585, 586, 588–588.9	2
Any malignancy including leukemia and lymphoma	140–172.9, 174–195.8, 200–208.9	2
Moderate or severe liver disease	572.2–572.8	3
Metastatic solid tumor	196–199.1	6
AIDS	042–044.9	6

The outcomes

The primary outcome of interest was to determine if opioid abuse among patients hospitalized for migraine, TTH, or CH during 2008-2014 was associated with differences in morbidity, disability, or discharge disposition. Morbidity was defined as patients transferred to a short-term hospital (STH), or skilled nursing facility (SNF), or intermediate care facility (ICF) and a hospital stay of more than eight days (>90th percentile of mean headache hospitalizations). The comparison of disability/loss of function was investigated by All Patient Refined Diagnosis Related Group (APR-DRG) severity between patients with opioid abuse and patients without opioid abuse. APR-DRGs were assigned using software developed by 3M Health Information Systems (Salt Lake City, UT), where score 0 indicates no loss of function, 1 indicates minor, 2 moderate, 3 major, and 4 indicates extreme loss of function. Detailed information on APR-DRGs is available at https://hcup-us.ahrq.gov/db/vars/aprdrg_severity/nisnote.jsp. Our secondary outcome of interest was to evaluate whether opioid-dependent and non-dependent opioid abuse was associated with headache disorders among patients hospitalized in the US between January 2013 and December 2014. The reason to choose the year 2013-2014 data for secondary outcome was a large number of US hospitalizations (more than 20 million) each year to evaluate patients with and without opioid abuse and headache disorders.

Statistical analysis

All statistical analyses were performed using the weighted survey methods in SAS version 9.4 (SAS Institute Inc., Cary, NC). Weighted values of patient-level observations were generated to produce a nationally representative estimate of the entire US population of hospitalized patients. A p-value of <0.05 was considered significant. Univariate analysis of differences between categorical variables was tested using the chi-square test, and analysis of differences between a continuous variable (age) was tested using paired Student's t-test. Mixed-effects survey logistic regression models with weighted analysis were used for categorical dependent variables to estimate the odds ratio (OR) and 95% confidence intervals for the association between opioid use and outcomes of interest among headache disorders from January 2008 to December 2014 and for the linkage between opioid use and headache disorders from January 2013 to December 2014.

We adjusted models with demographics (age, gender, race), patient-level hospitalization variables (admission day, primary payer, admission type, median household income category), hospital-level variables (hospital region, teaching versus non-teaching hospital, hospital bedsize), and CCI.

For each model, the c-index (a measure of goodness of fit for binary outcomes in a logistic regression model) was calculated. All statistical tests used were two-sided, and p<0.05 was deemed statistically significant. No statistical power calculation was conducted prior to the study.

Data availability

The data that supports the findings of this study is publicly available from the Agency for Healthcare Research and Quality's HCUP-NIS. A raw analysis of the data will be however made available from the authors upon request and with permission from HCUP-NIS.

## Results

Disease hospitalizations

From 2008 to 2014, after excluding patients with age <18 years and admissions with missing data for age, gender, and ethnicity, we found a total of 5,627,936 hospitalizations with headache disorders. Out of them, 128,383 (2.28%) were opioid abusers, 3,098,542 (55.06%) had migraine, 113,332 (2.01%) had TTH, and 26,572 (0.47%) had CH.

Trends and prevalence

**Figure 1:**
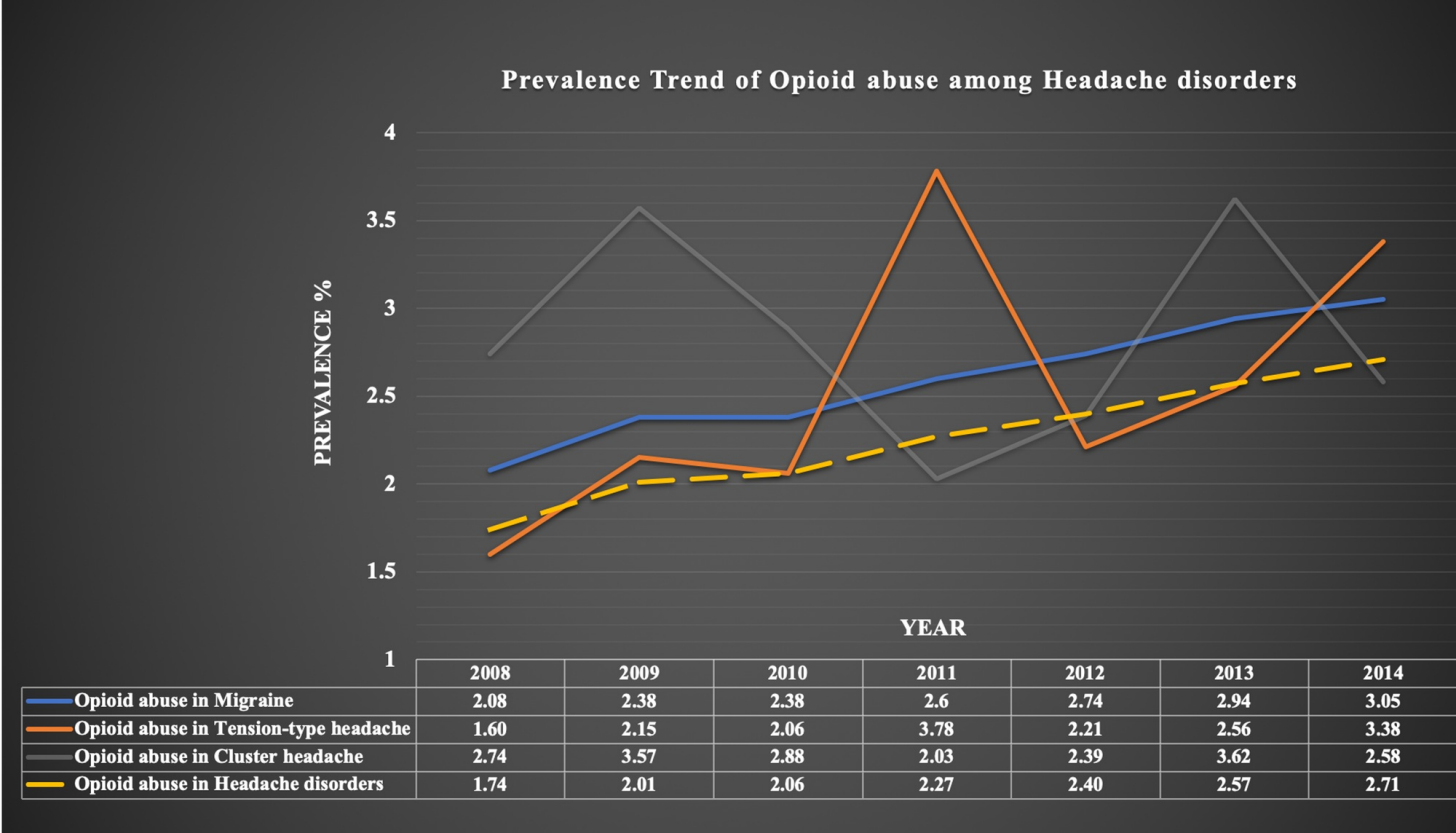
Trends of opioid abuse among patients with headache disorders

We analyzed trends of opioid abuse in total headache hospitalizations as well as in hospitalizations due to migraine, TTH, and CH. As shown in Figure 1, trends of opioid abuse were increasing from 2008 to 2014 in headache hospitalizations (1.74% in 2008 to 2.71% in 2014; p-trend<0.0001). We also found increased opioid abuse trends from 2008 to 2014 in migraine (2.08% in 2008 to 3.05% in 2014; p-trend<0.0001), TTH (1.60% in 2008 to 3.38% in 2014; p-trend<0.0001), and CH (2.74% in 2008 to 3.62% in 2013 and 2.58% in 2014; non-significant p-trend=0.3821). The opioid abusers had higher prevalence of migraine [81,573 (63.54%) vs. 3,016,969 (54.86%); p<0.0001], TTH [2943 (2.29%) vs. 110,389 (2.01%); p<0.0001], and CH [753 (0.59%) vs. 25,819 (0.47%); p<0.0001] compared to non-abusers (Table 2).

**Table 2:** Characteristics of opioid abusers among patients with primary headache disorders CCI, Charlson comorbidity index. Percentages in parentheses are column % indicating a direct comparison between opioid abusers and opioid non-abusers among patients with headache disorders. *Bedsize of hospital indicates the number of hospital beds that varies depending on hospital location (rural/urban), teaching status (teaching/non-teaching), and region (Northeast/Midwest/Southern/Western).

	Opioid abusers	Opioid non-abusers	Total	p-value
US hospitalization weighted, n (%)	128,383 (2.28)	5,499,553 (97.72)	5,627,936 (100)	<0.0001
Migraine	81,573 (63.54)	3,016,969 (54.86)	3,098,542 (55.06)	<0.0001
Tension-type headache	2943 (2.29)	110,389 (2.01)	113,332 (2.01)	<0.0001
Cluster headache	753 (0.59)	25,819 (0.47)	26,572 (0.47)	<0.0001
Demographics of patients				
Age median (SD) (years)	43 (13)	50 (17)		
Age group (years), n (%)				<0.0001
18-34	41,421 (32.26)	1,141,799 (20.76)	1,183,220 (21.02)	
35-49	45,403 (35.37)	1,591,363 (28.94)	1,636,766 (29.08)	
50-64	34,622 (26.97)	1,616,162 (29.39)	1,650,784 (29.33)	
65-79	6294 (4.90)	835,166 (15.19)	841,460 (14.95)	
≥80	644 (0.50)	315,064 (5.73)	315,708 (5.61)	
Gender, n (%)				<0.0001
Male	38,739 (30.17)	1,362,635 (24.78)	1,401,374 (24.90)	
Female	89,644 (69.83)	4,136,766 (75.22)	4,226,410 (75.10)	
Race, n (%)				<0.0001
White	101,681 (80.83)	3,921,186 (73.29)	4,022,867 (73.46)	
African American	14,266 (11.34)	791,546 (14.79)	805,812 (14.71)	
Hispanic	8269 (6.57)	518,840 (9.70)	527,109 (9.63)	
Asian or Pacific Islander	655 (0.52)	84,835 (1.59)	85,490 (1.56)	
Native American	929 (0.74)	34,090 (0.64)	35,019 (0.64)	
Characteristics of patients				
Median household income category for patient's Zip code, n (%)				0.0038
0-25th percentile	35,622 (28.62)	1,515,526 (28.18)	1,551,148 (28.19)	
26th-50th percentile	31,671 (25.45)	1,380,674 (25.67)	1,412,345 (25.67)	
51st-75th percentile	30,476 (24.49)	1,317,011 (24.49)	1,347,487 (24.49)	
76th-100th percentile	26,691 (21.45)	1,164,911 (21.66)	1,191,602 (21.66)	
Primary payer, n (%)				<0.0001
Medicare	34,266 (26.74)	1,716,332 (31.28)	1,750,598 (31.17)	
Medicaid	38,639 (30.15)	934,375 (17.03)	973,014 (17.33)	
Private insurance	35,039 (27.34)	2,209,402 (40.26)	2,244,441 (39.97)	
Other/self-pay/no charge	20,224 (15.78)	627,538 (11.44)	647,762 (11.53)	
Admission type, n (%)				<0.0001
Non-elective	109,357 (85.40)	4,305,363 (78.51)	4,414,720 (78.66)	
Elective	18,689 (14.60)	1,178,689 (21.49)	1,197,378 (21.34)	
Admission day, n (%)				<0.0001
Weekday	100,018 (77.91)	4,411,250 (80.21)	4,511,268 (80.16)	
Weekend	28,365 (22.09)	1,088,294 (19.79)	1,116,659 (19.84)	
Characteristics of hospitals				
Bedsize of hospital, n (%)*				<0.0001
Small	16,580 (12.98)	722,460 (13.23)	739,040 (13.22)	
Medium	33,110 (25.93)	1,374,792 (25.17)	1,407,902 (25.19)	
Large	78,004 (61.09)	3,365,141 (61.61)	3,443,145 (61.59)	
Hospital location and teaching status, n (%)				<0.0001
Rural	9750 (7.64)	548,194 (10.04)	557,944 (9.98)	
Urban non-teaching	52,173 (40.86)	2,111,699 (38.66)	2,163,872 (38.71)	
Urban teaching	65,771 (51.51)	2,802,500 (51.31)	2,868,271 (51.31)	
Hospital region, n (%)				<0.0001
Northeast	28,729 (22.38)	1,016,819 (18.49)	1,045,548 (18.58)	
Midwest	23,461 (18.27)	1,133,058 (20.60)	1,156,519 (20.55)	
South	45,175 (35.19)	2,277,278 (41.41)	2,322,453 (41.27)	
West	31,018 (24.16)	1,072,398 (19.50)	1,103,416 (19.61)	
Deyo's CCI, n (%)				<0.0001
0	71,861 (55.97)	2,634,272 (47.90)	2,706,133 (48.08)	
1	33,153 (25.82)	1,470,737 (26.74)	1,503,890 (26.72)	
2	11,878 (9.25)	688,501 (12.52)	700,379 (12.44)	
3	5250 (4.09)	307,168 (5.59)	312,418 (5.55)	
4	2338 (1.82)	153,580 (2.79)	155,918 (2.77)	
≥5	3904 (3.04)	245,294 (4.46)	249,198 (4.43)	

Demographics, patient and hospital characteristics, and comorbidities

Opioid abuse was more common in the 18-49 years of age group (p<0.0001). The individuals with opioid abuse were more likely to be males (30.17% vs. 24.78%; p<0.0001), white (80.83% vs. 73.29%; p<0.0001), Medicaid users (30.15% vs. 17.03%; p<0.0001), and non-elective admissions (85.40% vs. 78.51%; p<0.0001) compared to individuals without opioid abuse.

The outcomes

Table 3 mentions outcomes of opioid abusers among patients with headache hospitalizations. The overall morbidity was higher in opioid abusers (4.06% vs 3.70%; p<0.0001) than opioid non-abusers. Among headache hospitalizations, there was a higher prevalence of major/severe loss of function in opioid abusers compared to opioid non-abusers (28.14% vs. 22.43%; p<0.0001). There was a higher prevalence of opioid abusers who were transferred to short-term hospitalization (2.01% vs. 1.75%; p<0.0001) and transferred to a skilled nursing facility or intermediate care facility (8.84% vs. 7.33%; p<0.0001).

**Table 3:** Univariate analysis of outcomes of opioid abusers among patients with primary headache disorders APR-DRG, All Patients Refined Diagnosis Related Group; STH, short-term hospital, SNF, skilled nursing facility; ICF, intermediate care facility; SE, standard error. Percentages in parentheses are column % indicating a direct comparison between opioid abusers and opioid non-abusers among patients with headache disorders. *Morbidity: length of stay >8 days (>90 percentile of mean headache hospitalizations) and discharge other than home (STH, SNF, or ICF).

	Opioid abusers	Opioid non-abusers	Total	p-value
Morbidity, n (%)*	5210 (4.06)	203,567 (3.70)	208,777 (3.71)	<0.0001
APR-DRG severity or disability/loss of function, n (%)				<0.0001
No loss of function	≤10	825 (0.02)	835 (0.01)	
Minor loss of function	24,462 (19.14)	1,704,852 (31.14)	1,729,314 (30.87)	
Moderate loss of function	67,376 (52.71)	2,541,186 (46.42)	2,608,562 (46.56)	
Major loss of function	30,579 (23.92)	1,077,846 (19.69)	1,108,425 (19.78)	
Severe loss of function	5392 (4.22)	150,151 (2.74)	155,543 (2.78)	
Total major/severe loss of function (%)	35,971 (28.14)	1,227,997 (22.43)	1,263,968 (22.56)	<0.0001
Discharge disposition, n (%)				<0.0001
Routine/home	100,831 (82.87)	4,406,565 (81.59)	4,507,396 (81.62)	
Transfer to STH	2444 (2.01)	94,652 (1.75)	97,096 (1.76)	
Transfer to SNF/ICF/another type of facility	10,752 (8.84)	396,005 (7.33)	406,757 (7.37)	
Home health care	7644 (6.28)	503,787 (9.33)	511,431 (9.26)	
Total discharge other than home	20,840 (17.13)	994,443 (18.41)	1,015,283 (18.38)	<0.0001

Regression model derivation

We performed the multivariable survey logistic regression models to predict the outcomes of opioid abusers (morbidity, disability, and discharge disposition) among patients with headache disorders after adjusting for basic demographic characteristics with patient and hospital-level variables, and CCI (Table 4). In this multivariate regression analysis, opioid abusers had higher odds of morbidity (adjusted OR [aOR]: 1.48; 95% CI: 1.39-1.59; p<0.0001) compared to opioid non-abusers. Similarly, opioid abusers also had higher odds of major/severe disability (aOR: 1.58; 95% CI: 1.53-1.63; p<0.0001), and discharge to short-term hospital or skilled nursing facility or intermediate care facility (aOR: 1.35; 95% CI: 1.30-1.40; p<0.0001) compared to opioid non-abusers (Table 4).

**Table 4:** Multivariable logistic regression analysis to predict outcomes of opioid abusers among patients with primary headache disorders OR, odds ratio; CCI, Charlson comorbidity index; STH, short-term hospital, SNF, skilled nursing facility; ICF, intermediate care facility; APR-DRG, All Patients Refined Diagnosis Related Group *Morbidity was defined as length of stay >8 days (>90 percentile of mean headache hospitalizations) and discharge other than home (STH, SNF, or ICF). ^†^Disability was defined by major/severe APR-DRG loss of function on discharge. ^‡^Discharge disposition/outcome was defined as home versus non-home (STH, SNF, or ICF).

	Model 1: odds of morbidity*	Model 2: odds of major or severe disability^†^	Model 3: odds of non-home discharge disposition^‡^
	OR; CI; p-value	OR; CI; p-value	OR; CI; p-value
No opioid abuse	Reference		
Opioid abuse	1.48; 1.39-1.59; <0.0001	1.58; 1.53-1.63; <0.0001	1.35; 1.30-1.40; <0.0001
Age (every 10 years)	1.02; 1.02-1.02; <0.0001	1.00; 1.00-1.00; <0.0001	1.04; 1.04-1.04; <0.0001
Gender			
Female	Reference		
Male	1.16; 1.14-1.19; <0.0001	1.12; 1.11-1.13; <0.0001	0.95; 0.94-0.96; <0.0001
Race			
White	Reference		
African American	0.97; 0.94-1.00; 0.0792	0.92; 0.91-0.94; <0.0001	0.94; 0.92-0.95; <0.0001
Hispanic	0.90; 0.86-0.93; <0.0001	0.82; 0.80-0.83; <0.0001	0.77; 0.76-0.79; <0.0001
Asian or Pacific Islander	1.10; 1.02-1.19; 0.0163	0.92; 0.88-0.96; <0.0001	0.82; 0.78-0.85; <0.0001
Native American	0.86; 0.73-1.00; 0.0479	0.92; 0.87-0.98; 0.0118	0.84; 0.78-0.90; <0.0001
Median household income category for patient's Zip code			
0-25th percentile	Reference		
26th-50th percentile	0.99; 0.96-1.02; 0.5295	1.01; 0.99-1.02; 0.2527	0.99; 0.98-1.01; 0.2400
51st-75th percentile	0.97; 0.94-0.99; 0.0201	1.04; 1.02-1.05; <0.0001	0.99; 0.97-1.00; 0.0626
76th-100th percentile	0.95; 0.92-0.98; 0.0030	1.05; 1.04-1.07; <0.0001	0.99; 0.98-1.01; 0.4194
Primary payer			
Medicare	Reference		
Medicaid	0.85; 0.82-0.88; <0.0001	0.81; 0.79-0.82; <0.0001	0.77; 0.75-0.78; <0.0001
Private insurance	0.63; 0.61-0.65; <0.0001	0.66; 0.65-0.67; <0.0001	0.60; 0.59-0.61; <0.0001
Other/self-pay/no charge	0.51; 0.48-0.53; <0.0001	0.61; 0.59-0.62; <0.0001	0.49; 0.48-0.50; <0.0001
Admission type			
Non-elective	Reference		
Elective	1.40; 1.37-1.44; <0.0001	0.63; 0.62-0.64; <0.0001	1.59; 1.57-1.61; <0.0001
Admission day			
Weekday	Reference		
Weekend	1.10; 1.07-1.13; <0.0001	1.06; 1.04-1.07; <0.0001	1.00; 0.99-1.01; 0.8087
Bedsize of hospital			
Small	Reference		
Medium	1.17; 1.31-1.22; <0.0001	1.09; 1.07-1.10; <0.0001	0.98; 0.96-1.00; 0.0188
Large	1.46; 1.41-1.51; <0.0001	1.19; 1.17-1.21; <0.0001	0.97; 0.96-0.99; 0.0006
Hospital location and teaching status			
Rural	Reference		
Urban non-teaching	1.49; 1.42-1.55; <0.0001	1.11; 1.09-1.13; <0.0001	1.09; 1.06-1.11; <0.0001
Urban teaching	1.86; 1.79-1.94; <0.0001	1.42; 1.40-1.45; <0.0001	1.09; 1.07-1.11; <0.0001
Hospital region			
Northeast	Reference		
Midwest	0.88; 0.86-0.91; <0.0001	1.42; 1.40-1.44; <0.0001	0.76; 0.75-0.77; <0.0001
South	0.85; 0.83-0.88; <0.0001	1.41; 1.39-1.43; <0.0001	0.69; 0.68-0.70; <0.0001
West	0.81; 0.78-0.84; <0.0001	1.59; 1.57-1.62; <0.0001	0.70; 0.69-0.71; <0.0001
Deyo's CCI	1.24; 1.23-1.24; <0.0001	1.54; 1.54-1.55; <0.0001	1.18; 1.17-.1.18; <0.0001
c-index	0.73	0.73	0.74

Accuracy of the model

Adjusted models to predict morbidity, disability, and discharge disposition had the c-statistic of 0.73, 0.73, and 0.74, respectively, which are >0.7 indicating a good model.

Analysis to predict the linkage between headache disorders and opioid abuse from January 2013 to December 2014

From January 2013 to December 2014, a total of 56,581,503 hospitalizations were analyzed. Among them, 978,860 (1.73%) patients had a history of opioid abuse. There was a higher prevalence of opioid abuse among migraineurs compared to non-migraineurs (3.00% vs. 1.71%; p<0.0001), patients with TTH compared to without TTH (2.97% vs. 1.73%; p<0.0001), and patients with CH compared to without CH (3.10% vs. 1.73%; p<0.0001) (Table 5).

**Table 5:** Univariate analysis of the linkage between primary headache disorders and opioid abuse

Linkage between primary headache disorders and opioid abuse	
Migraine vs. no migraine among opioid abusers	29485 (3.00%) vs. 949375 (1.71%) (p<0.0001)
Tension-type headache vs. no tension-type headache among opioid abusers	960 (2.97%) vs. 977900 (1.73%) (p<0.0001)
Cluster headache vs. no cluster headache among opioid abusers	275 (3.10%) vs. 978585 (1.73%) (p<0.0001)

In the regression analysis, after adjusting for basic demographics with the patients and hospital-level variables, and CCI, patients with migraine (aOR: 1.61; 95% CI: 1.57-1.66; p<0.0001), TTH (aOR: 1.43; 95% CI: 1.22-1.66; p<0.0001), and CH (aOR: 1.34; 95% CI: 1.01-1.78; p=0.0421) were having higher odds of exposure to opioid abuse than patients without these headache disorders (Table 6). The c-statistic was 0.78, which indicates a good model.

**Table 6:** Adjusted multivariable logistic regression analysis to predict the linkage between primary headache disorders and opioid abuse OR, odds ratio; UL, upper limit; LL, lower limit; CCI, Charlson comorbidity index

	OR	CI		p-value
		LL	UL	
No migraine	Reference			
Migraine	1.61	1.57	1.66	<0.0001
No tension-type headache	Reference			
Tension-type headache	1.43	1.22	1.66	<0.0001
No cluster headache	Reference			
Cluster headache	1.34	1.01	1.78	0.0421
Age (every 10 years)	0.96	0.96	0.96	<0.0001
Gender				
Female	Reference			
Male	1.92	1.90	1.94	<0.0001
Race				
White	Reference			
African American	0.59	0.58	0.60	<0.0001
Hispanic	0.38	0.38	0.39	<0.0001
Asian or Pacific Islander	0.16	0.15	0.17	<0.0001
Native American	0.76	0.72	0.80	<0.0001
Median household income category for patient's Zip code				
0-25th percentile	Reference			
26th-50th percentile	0.81	0.80	0.82	<0.0001
51st-75th percentile	0.79	0.78	0.80	<0.0001
76th-100th percentile	0.77	0.76	0.79	<0.0001
Primary payer				
Medicare	Reference			
Medicaid	1.72	1.69	1.75	<0.0001
Private insurance	0.54	0.53	0.55	<0.0001
Other/self-pay/no charge	1.41	1.38	1.44	<0.0001
Admission type				
Non-elective	Reference			
Elective	0.53	0.52	0.54	<0.0001
Admission day				
Weekday	Reference			
Weekend	1.03	1.02	1.04	<0.0001
Bedsize of hospital				
Small	Reference			
Medium	0.91	0.90	0.93	<0.0001
Large	0.90	0.89	0.91	<0.0001
Hospital location and teaching status				
Rural	Reference			
Urban non-teaching	1.40	1.37	1.42	<0.0001
Urban teaching	1.43	1.40	1.45	<0.0001
Hospital region				
Northeast	Reference			
Midwest	0.61	0.60	0.62	<0.0001
South	0.49	0.48	0.49	<0.0001
West	0.76	0.75	0.77	<0.0001
Deyo's CCI	0.94	0.94	0.95	<0.0001
c-index	0.78			

## Discussion

Headache is the most common neurological disorder presenting to primary care, accounting for 3% of all visits [9]. In 2007, Stovner et al. reported a global prevalence of 46% for headache in general, 11% for migraine, 42% for tension-type headache, and 3% for chronic daily headache [10]. In 2015, the Global Burden of Disease estimated that headache disorders including their subtypes - migraine, TTH, and CH - were the third cause of disability in people less than 50 years of age [11]. Opioid abuse is of particular concern in headache disorders given its role as a treatment modality. Over the last two decades, the opioid epidemic has led to enormous health concerns and it has been triggered to a large extent by prescription opioids: an 80% increase in opioid analgesic prescriptions from the year 2000 through 2010 with incremental use from 7.4% to 11.8% [12]. Similarly, in our study, we found that among the adult American population, opioid use increased from 1.74% in 2008 to 2.71% in 2014.

In recent years, there has been a tremendous increase in opioid prescriptions for acute and chronic pain and this trend is also seen in patients with headache disorders [13]. The American Migraine Prevalence and Prevention Study (AMPP) involving 6008 migraine patients reported that 16.6% of patients met Diagnostic and Statistical Manual of Mental Disorders, Fourth Edition (DSM-IV) criteria for opioid dependence [14]. A study by Choong et al. reported that the most common medicine prescribed for cluster headache is opioids [15]. Our study found that among 5,627,936 headache hospitalizations, 128,383 (2.28%) were opioid abusers. We also found a significant increasing trend of opioid abuse (years 2008-2014) among headache hospitalizations (1.74% in 2008 to 2.71% in 2014; p-trend<0.0001). This recent trend in overprescribing opioids for various headache disorders despite the lack of strong evidence showing the efficacy of opioid treatment has led to serious consequences both affecting an individual's quality of life and increased burden on society and healthcare that must now be addressed conscientiously.

Opioid use can also lead to significantly higher disability in patients admitted for headaches. Our study also found 1.48 times higher odds of morbidity and 1.58 times higher odds of major/severe disability among opioid abusers compared to opioid non-abusers (p<0.0001). This increased disability can be due to chronic opioid therapy in treating chronic migraine and as prophylaxis for refractory headache. Considering the risks including disability, low quality of life, and higher healthcare costs associated with opioid abuse and lack of opioid efficacy data in the treatment of migraine, it is crucial for providers to evaluate potential benefits of alternate treatment options. Several guidelines have been published for the safe use of opioid medications in the treatment of chronic pain that emphasize screening prior to prescribing opioids [16,17].

To prevent opioid abuse and its associated adverse effects, it is crucial that the patient's medication regimen is reviewed at each visit as well as screening for impaired cognition, use of other illicit or prescription drugs, and concurrent mental illness has been done as all of these factors may increase the risk of opioid overdose [18]. Among patients treated with chronic opioids, a urine toxicology screen should be done at the first clinic visit and then annually to assess for potential polysubstance abuse [19].

Strength and limitations

To our knowledge, this is the first large population-based nationwide study to report prevalence, outcomes, and linkage between opioid abuse and headache disorders. One of the limitations of this study being an observational study is that we cannot prove causation of the temporal association of opioids for headache disorders. Also, our assessment is limited to hospitalized patients with headaches and may not reflect the severity of the issue in outpatients. Long-term outcomes are also not available through this study. In spite of these limitations, we have a very large number of patients in the study, which is possible through the use of a nationwide database such as NIS. The APR-DRG coding system used in this study to assess the severity of illness is an external validated reliable method with accurate and consistent results and is widely used by hospitals [20,21]. A population-wide study with a large number of subjects is ideally suited to understand the impact of the opioid epidemic on headache disorders.

## Conclusions

We have found that opioid abusers were associated with the higher prevalence of migraine, TTH, and CH and also had higher odds of morbidity, severe disability, and non-home discharge as compared to non-abusers. The patients with these primary headache disorders were having higher odds of exposure to opioid abuse than patients without these headache disorders. A careful selection of patients for opioid prescription and refill, counselling for recreational use, and identification of such patients might mitigate the risk of opioid abuse-associated poor outcomes among patients with headache disorders.
